# Endoscopic omental packing in combination with the clip-and-snare method using pre-looping technique for gastric perforation

**DOI:** 10.1055/a-2097-9037

**Published:** 2023-06-15

**Authors:** Naohiro Yoshida, Manami Utsunomiya, Azusa Kawasaki, Hiroyoshi Nakanishi, Shigetsugu Tsuji, Kenichi Takemura, Hisashi Doyama

**Affiliations:** Department of Gastroenterology, Ishikawa Prefectural Central Hospital, Ishikawa, Japan

Endoscopic omental packing (EOP) is a technique used to close gastric perforations and is particularly useful for large defects 1 . We developed the pre-looping technique (PLT) as a simple and safe method of snare delivery into the stomach, and reported applications of the clip-and-snare method using PLT (CSM-PLT) as a traction method for endoscopic submucosal dissection (ESD) 2 3 . We hereby describe a case where CSM-PLT was used for EOP during intraoperative perforation of gastric ESD.

Perforation occurred during ESD for gastric cancer in the greater curvature of the lower body. Although conventional EOP was performed ( Fig. 1 ) and subsequent ESD was completed, the perforation reopened immediately after removal of the lesion ( Fig. 2 ). We performed EOP using CSM-PLT as follows. The endoscope was withdrawn and the snare (SD-221U-25; Olympus, Tokyo, Japan) was looped around the transparent cap on the outside of the endoscope ( Fig. 3 ); the endoscope with snare attached was then reinserted. A hemostatic clip (HX-610-090; Olympus) was inserted through the endoscope channel and used to grasp the omentum, taking care not to completely release the clip from the deployment device. The pre-looped snare was then loosened from the transparent cap and moved along the device toward the hemostatic clip, which was then grasped with the snare and released from the deployment device. The snare was used independently of the endoscope to apply the appropriate tension to the omentum and firmly attach it to the stomach wall using clips. Once traction was no longer required, the snare was loosened and removed ( Fig. 4 , Video 1 ).

**Fig. 1:**
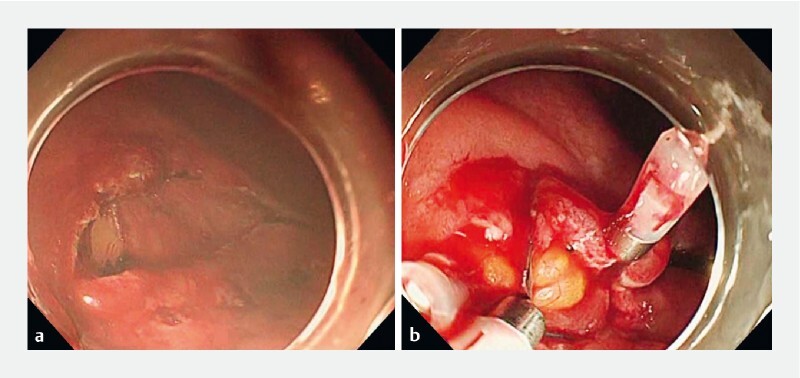
Conventional endoscopic omental packing for gastric perforation during endoscopic submucosal dissection. **a** Perforation during circumferential mucosal incision. **b** Conventional endoscopic omental packing.

**Fig. 2:**
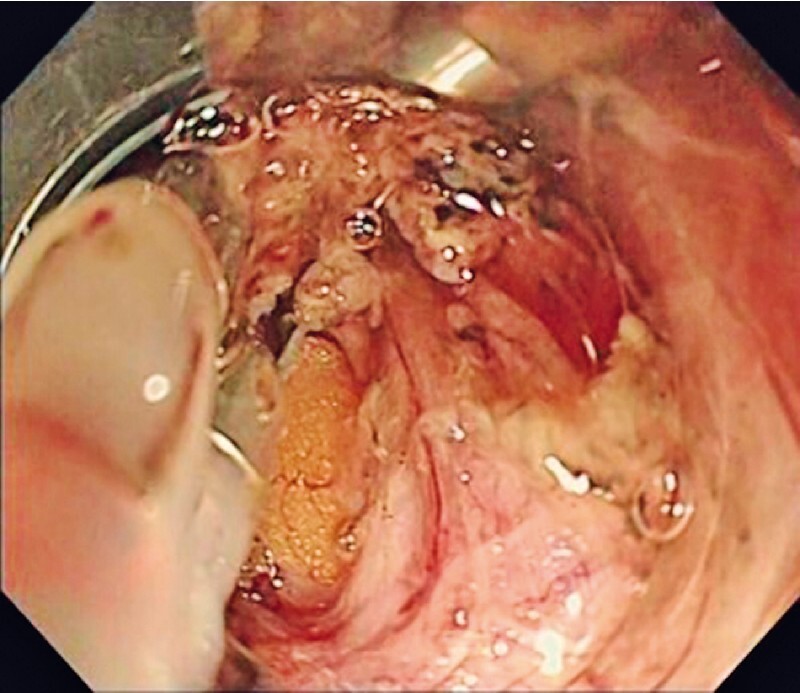
The perforation reopened after removal of the lesion.

**Fig. 3:**
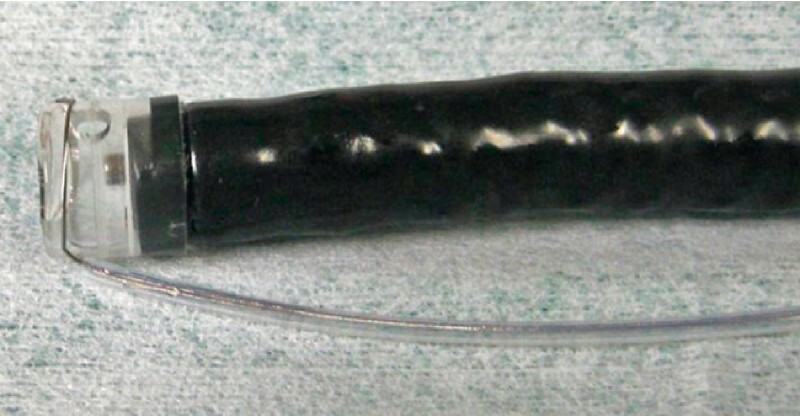
Pre-looping technique. The transparent cap on the endoscope tip was tightened using the snare located on the outside of the endoscope.

**Fig. 4:**
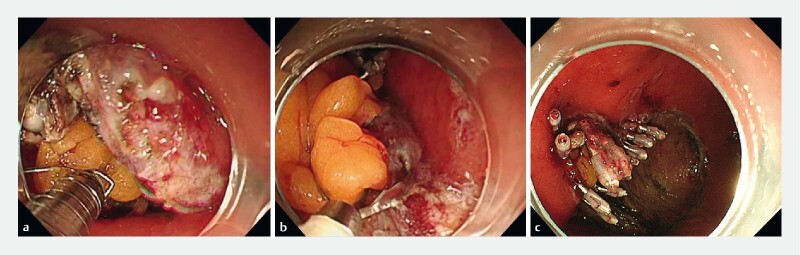
Endoscopic omental packing in combination with the clip-and-snare method using pre-looping technique (CSM-PLT). **a** After grasping the omentum using the hemostatic clip, the pre-looped snare was loosened from the transparent cap and used to grasp the hemostatic clip. **b** The omentum, adequately tensioned by CSM-PLT, was clipped to the stomach wall. **c** Complete closure was achieved.

**Video 1** Endoscopic omental packing in combination with the clip-and-snare method using pre-looping technique for gastric perforation.


The patient’s subsequent clinical course was good and scarring of the post-ESD ulcer was confirmed ( Fig. 5 ).

**Fig. 5:**
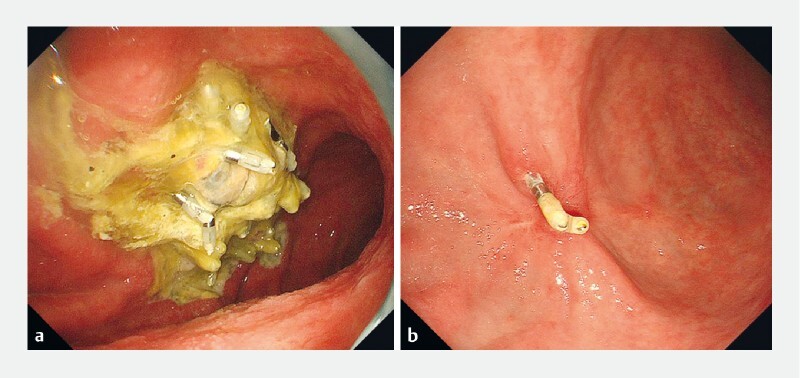
Scarring of the ulcer after endoscopic submucosal dissection (ESD): **a** 1 week after ESD. **b** 6 months after ESD.

CSM-PLT represents a simple and reliable method for EOP. Previous reports demonstrated the effectiveness of EOP for gastric wall closure after endoscopic full-thickness resection 4 5 ; we propose that CSM-PLT is also applicable in such cases.

Endoscopy_UCTN_Code_CPL_1AH_2AZ

## Notes

Endoscopy E-Videos

https://eref.thieme.de/e-videos
*E-Videos* is an open access online section of the journal *Endoscopy* , reporting on interesting cases and new techniques in gastroenterological endoscopy. All papers include a high-quality video and are published with a Creative Commons CC-BY license. Endoscopy E-Videos qualify for HINARI discounts and waivers and eligibility is automatically checked during the submission process. We grant 100% waivers to articles whose corresponding authors are based in Group A countries and 50% waivers to those who are based in Group B countries as classified by Research4Life (see: https://www.research4life.org/access/eligibility/ ). This section has its own submission website at https://mc.manuscriptcentral.com/e-videos
